# An itinerant antiferromagnetic metal without magnetic constituents

**DOI:** 10.1038/ncomms8701

**Published:** 2015-07-13

**Authors:** E. Svanidze, Jiakui K. Wang, T. Besara, L. Liu, Q. Huang, T. Siegrist, B. Frandsen, J. W. Lynn, Andriy H. Nevidomskyy, Monika B. Gamża, M. C. Aronson, Y. J. Uemura, E. Morosan

**Affiliations:** 1 Department of Physics and Astronomy, Rice University, Houston, Texas 77005, USA; 2 National High Magnetic Field Laboratory, Florida State University, Tallahassee, Florida 32306, USA; 3 Department of Physics, Columbia University, New York, New York 10027, USA; 4 NIST Center for Neutron Research, National Institute of Standards and Technology, Gaithersburg, Maryland 20899, USA; 5 Condensed Matter Physics and Materials Science Department, Brookhaven National Laboratory, Upton, New York 11973, USA; 6 Department of Physics and Astronomy, Stony Brook University, Stony Brook, New York 11794, USA

## Abstract

The origin of magnetism in metals has been traditionally discussed in two diametrically opposite limits: itinerant and local moments. Surprisingly, there are very few known examples of materials that are close to the itinerant limit, and their properties are not universally understood. In the case of the two such examples discovered several decades ago, the itinerant ferromagnets ZrZn_2_ and Sc_3_In, the understanding of their magnetic ground states draws on the existence of 3*d* electrons subject to strong spin fluctuations. Similarly, in Cr, an elemental itinerant antiferromagnet with a spin density wave ground state, its 3*d* electron character has been deemed crucial to it being magnetic. Here, we report evidence for an itinerant antiferromagnetic metal with no magnetic constituents: TiAu. Antiferromagnetic order occurs below a Néel temperature of 36 K, about an order of magnitude smaller than in Cr, rendering the spin fluctuations in TiAu more important at low temperatures. This itinerant antiferromagnet challenges the currently limited understanding of weak itinerant antiferromagnetism, while providing insights into the effects of spin fluctuations in itinerant–electron systems.

## Abstract (web summary)

Sc_3_In and ZrZn_2_ are the only two known itinerant ferromagnets that form from non-magnetic constituents. Now, Svanidze *et al.,* evidence itinerant antiferromagnetism in TiAu below 36 K using thermodynamic, transport, muon-based and neutron-based measurements, and density functional analysis.

## Graphical abstract

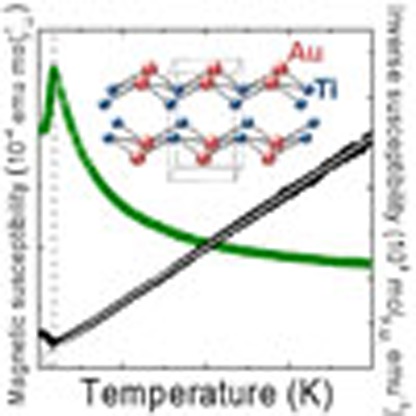

The local and itinerant moment are extreme limits of magnetic behaviour, with poorly understood physics associated with in-between scenarios. While the local moment magnetism (or real-space-localized magnetic moments and fluctuations) was readily understood early on within the Heisenberg model^1^ using a Weiss molecular field, the itinerant moment behaviour (corresponding to moments and fluctuations localized in the reciprocal space) can only partially be reproduced by current theoretical approaches. Despite the success of the Stoner model^2^ and the subsequent improvements when spin fluctuations were accounted for^3^,^4^,^5^, a unified picture of magnetism (which to encompass both extreme scenarios, local and itinerant moment) remains elusive. A practical limitation is the small number of known itinerant moment magnetic systems. In one extreme case, that of magnetic metals without magnetic constituents, only two itinerant ferromagnets (IFMs), Sc_3_In (ref. 6) and ZrZn_2_ (ref. 7), have been known for 50 years.

Here we report evidence for an itinerant antiferromagnet (IAFM) with no magnetic constituents, TiAu. Given the small number of known itinerant magnets, the discovery of the IAFM TiAu provides an opportunity for detailed studies which to advance the understanding of the physics of itinerant magnets in general, and of IAFMs in particular. Thermodynamic and transport measurements reveal the antiferromagnetic order at a Néel temperature *T*_N_≅36 K. The long-range order is confirmed by neutron diffraction data, while muon spin relaxation (μSR) experiments indicate static order. Together these measurements point to small moment ordering in the whole sample volume. In addition to the experimental evidence for the itinerant moment antiferromagnetic order in TiAu, density functional theory (DFT) calculations confirm the spin density wave (SDW) small moment ordering.

## Results

### Evidence for itinerant moment antiferromagnetic order

TiAu has been reported to form in three distinct crystal structures, cubic *Pm*
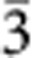
*m*^8^, tetragonal *P*4*/nmm*^9^ and orthorhombic *Pmma*^8^, posing an inherent difficulty in synthesizing it as a single phase. It comes as no surprise then, that no reports of physical properties of TiAu exist. Here we report the magnetic and electronic properties of phase-pure orthorhombic TiAu, and that it is an itinerant electron antiferromagnet.

**Figure 1:**
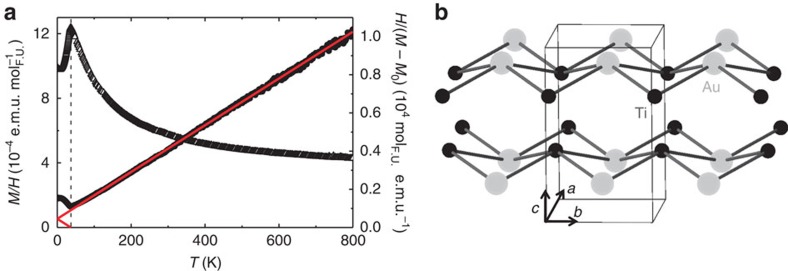
Temperature-dependent magnetization of TiAu. (**a**) Left axis: zero-field-cooled magnetic susceptibility as a function of temperature for *μ*_0_*H*=0.1 T applied field (1 e.m.u.=10 A cm^−2^). Right axis: inverse susceptibility *H*/*M* along with a Curie–Weiss-like fit (solid line), with *θ≈*−37 K (marked by a dashed line). (**b**) The crystal structure of TiAu with Ti (small) and Au (large) atoms, and the orthorhombic unit cell outlined in thin black lines. The arrows identify the crystallographic directions, marked by *a*, *b* and *c* axes.

The first evidence for the antiferromagnetic ground state is the cusp around 36 K in the temperature-dependent magnetic susceptibility *M*(*T*)/*H*, shown in Fig. 1 (left axis). By analogy with local moment AFMs, the TiAu zero-field-cooled and field-cooled data are indistinguishable. The value of the temperature-independent Pauli susceptibility, calculated from the magnetic density of states *χ*_0_≈0.2 × 10^−3^ e.m.u. mol^−1^, agrees well with the experimental one *M*_0_/*H*≈0.3 × 10^−3^ e.m.u. mol^−1^. Upon warming above the Néel temperature, the inverse susceptibility *H*/(*M*–*M*_0_) (right axis, Fig. 1) is linear in temperature up to 800 K. Such linear *T* dependence of *H*/*M* has been long considered the hallmark of local moment magnetism, until it was observed in the weak IFMs without local moments, ZrZn_2_ (ref. 7) and Sc_3_In (ref. 6). Puzzling at first, the origin of this behaviour was reconciled in the case of IFMs, when spin fluctuation effects were considered by Moriya^3^,^4^. The self-consistent renormalization theory unified the local and itinerant pictures of ferromagnetism, and postulated a new origin for the Curie–Weiss-like susceptibility in the latter, as the interactions of the spatially extended modes of spin fluctuations^10^,^11^. TiAu however is an IAFM, and no existing theory accounts for an IAFM ground state if neither Ti nor Au have conventional local moments. X-ray photoemission spectroscopy (XPS) analysis suggests that Ti is close to the non-magnetic 4+ oxidation state (Supplementary Note 1). This is unexpected in light of the high magnetic volume fraction observed in the μSR measurements presented below, which, together with the single phase XPS and neutron patterns in Supplementary Figs 1 and 2, indicates that the observed magnetic behaviour is indeed intrinsic. Therefore, this suggests that *Pmma* TiAu is the first IAFM metal with no magnetic constituents, with the magnetic ground state strongly affected by spin fluctuations.

**Figure 2:**
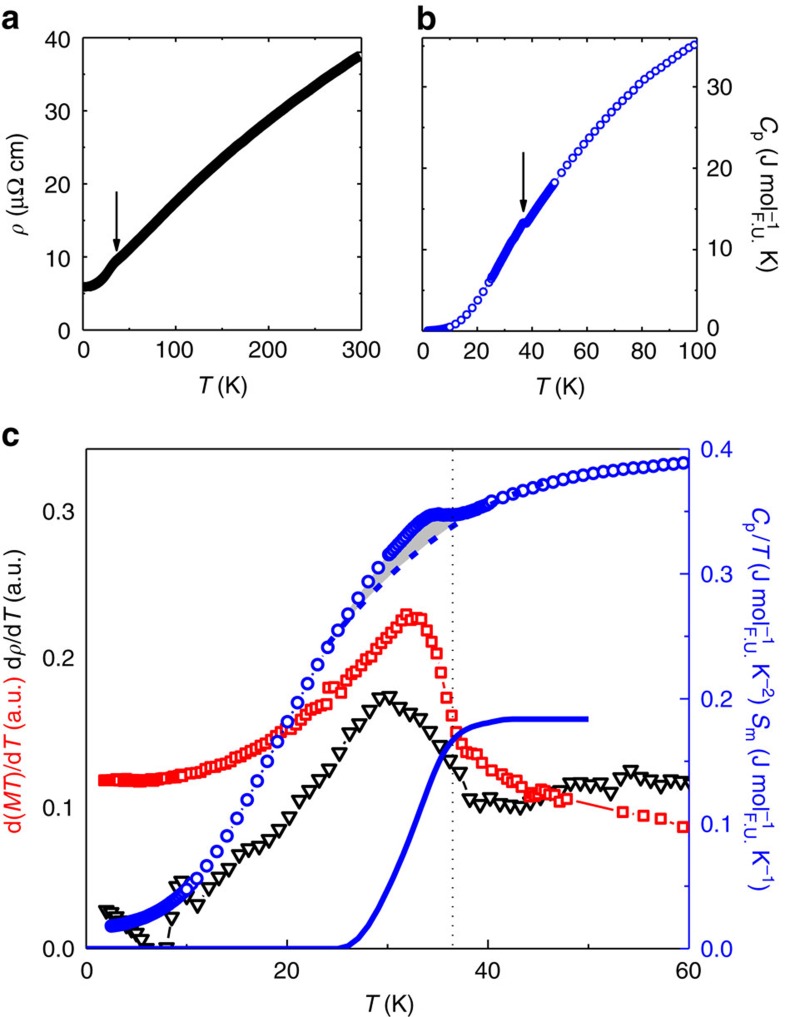
Specific heat and resistivity of TiAu. *H*=0 temperature-dependent resistivity (**a**) and specific heat (**b**) with black arrows indicating the feature corresponding to *T*_N_. (**c**) The ordering temperature *T*_N_ (vertical dotted line) for TiAu determined from peaks in the temperature derivatives of resistivity, d*ρ*/d*T* (*H*=0, black triangles, left axis), and d(*MT*)/d*T* (*μ*_0_*H*=0.1 T, red squares, left axis), and in *C*_p_/*T* (*H*=0, blue circles, right axis). The entropy *S*_m_ (solid blue line, right axis) is calculated by subtracting a polynomial non-magnetic component (dashed blue line) from the measured specific heat data (blue circles).

By contrast with most known weak itinerant (ferro- or antiferro-) magnets, the TiAu electrical resistivity (Fig. 2a) and specific heat data (Fig. 2b) also show signatures of the phase transition around 36 K. The *H*=0 *ρ*(*T*) data are typical of a good metal, decreasing nearly linearly from room temperature down to ∼40 K. A drop of about 10%, similar to the loss of spin-disorder scattering, occurs at *T*_N_ (Fig. 2a). Although often the gap opening associated with the SDW ordering results in a resistivity increase, a similar drop was observed in BaFe_2_As_2_ single crystals^12^. In the absence of local moment ordering, the decrease in the resistivity at *T*_N_ results from the balance of the loss of scattering due to Fermi surface nesting (see below) and the gap opening due to the SDW AFM state. At the same temperature in TiAu, a small peak becomes visible in the specific heat data *C*_p_ (Fig. 2b), such that *T*_N_ in this AFM metal can be determined, as shown by Fisher^13^,^14^, from peaks in *C*_p_ (most visible in *C*_p_/*T*), d(*MT*)d*T* and d*ρ*/d*T* (Fig. 2c). Distinguishing between local and itinerant moment magnetism is inherently difficult, especially in the nearly unexplored realm of IAFMs. It is therefore surprising that in TiAu, the evidence points toward its itinerant magnetic moment character. The fact that the peak in *C*_p_ is not as strong as Fisher's prediction^13^ is one such argument favouring the itinerant moment scenario in TiAu. Another argument is the small magnetic entropy *S*_m_ (grey area, Fig. 2c) associated with the transition (solid blue line, Fig. 2c). Even though the *S*_m_ calculated after assuming a polynomial non-magnetic *C*_p_ around the transition (dashed line, Fig. 2c) is an underestimate, it amounts to only 0.2 J mol^−1^ K^−1^ or ∼3% of *R*ln2.

**Figure 3:**
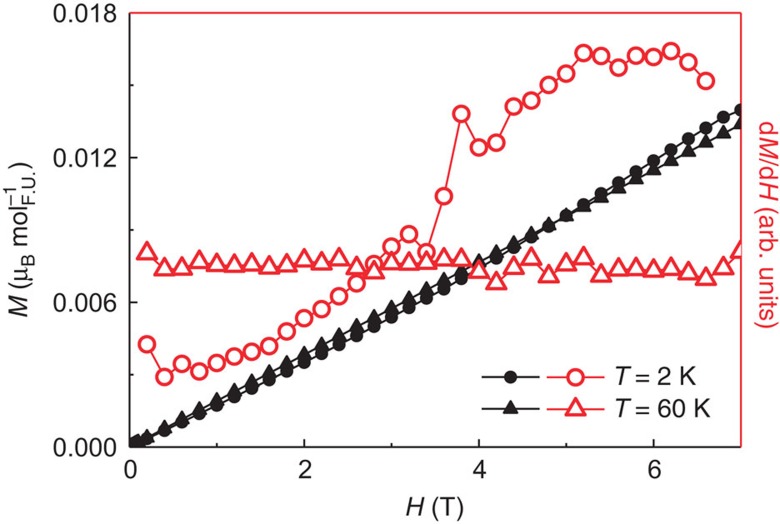
Field-dependent magnetization of TiAu. The magnetization isotherms *M*(*H*) (full symbols, left axis) and the derivative d*M*/d*H* (open symbols, right axis) for *T*=2 K (circles) and 60 K (triangles). No saturation is achieved for magnetic fields up to 7 T. A metamagnetic transition is observed around 4 T in the *T*=2 K isotherm, but not in the one above *T*_N_.

Despite the remarkably large paramagnetic moment *μ*_PM_≅0.8 μ_B_, derived from the Curie–Weiss-like fit of the inverse susceptibility (Fig. 1a, right axis), the field-dependent magnetization *M*(*H*) does not saturate up to *μ*_0_*H*=7 T, and the maximum measured magnetization is only 0.01 μ_B_ (Fig. 3). A closer look at the low temperature *M*(*H*) reveals a weak metamagnetic transition starting around *μ*_0_*H*=3.6 T for *T*=2 K (circles, Fig. 3). This is most apparent in the derivative d*M*/d*H* (open symbols) rather than in the as-measured isotherms (full symbols), with the latter nearly indistinguishable well below (*T*=2 K) and above (*T*=60 K) the magnetic ordering temperature. It has been shown by Sandeman *et al.*^15^, that, within the Stoner theory, the presence of a sharp double peak structure in the electronic density of states (DOS) sufficiently close to the Fermi level results in a metamagnetic transition. The argument requires that the paramagnetic Fermi level lie in between the two peaks of the DOS, and this is indeed revealed by the band structure calculations for TiAu, as is shown below. It results that, as the Fermi sea is polarized by the applied magnetic field *H*, the majority and minority spin Fermi levels feel the effect of the two DOS peaks at different values of induced magnetization. The DOS peak that is closest to the Fermi level will lead to a sharp increase (decrease) in the population of the majority (minority) spin band, resulting in a metamagnetic transition.

### Evidence of Static magnetic order in the full volume

**Figure 4:**
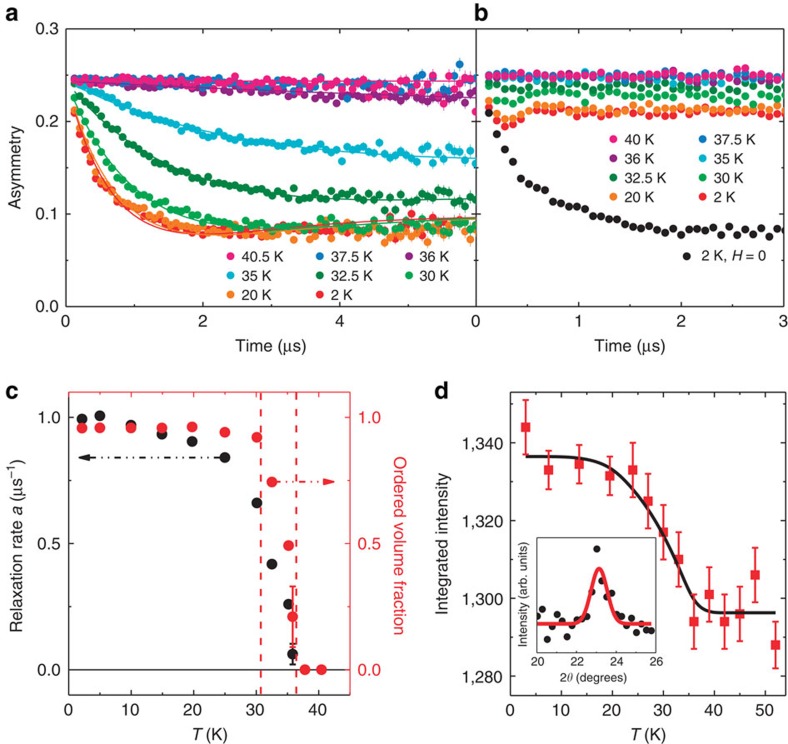
Muon spin relaxation and neutron diffraction. (**a**) Time dependence of the asymmetry in *H*=0, fit with equation (1) (solid lines). (**b**) A small applied longitudinal field *μ*_0_*H*=0.01 T eliminates the relaxation response. For comparison, the *H*=0 *T*=2 K asymmetry is shown in black circles. (**c**) Relaxation rate *a* (black circles, left) and volume fraction (red circles, right) as a function temperature (*H*=0). (**d**) Integrated intensity of the (0, *π*/*b*, 0) TiAu magnetic Bragg peak as a function of temperature with mean-field fit (*T*_N_=36±2 K, black curve). Inset: net counts normalized for 2 min. counting time between 2.5 and 60 K, showing the (0, *π*/*b*, 0) magnetic peak fit with a resolution-limited Gaussian (red line). Uncertainties are statistical in origin and represent one standard deviation.

Muon spin relaxation data shown in Fig. 4 support the static magnetic order developing in the full volume fraction, with the transition temperature corresponding to the anomaly in the magnetic susceptibility, resistivity and specific heat shown in Fig. 2c. For temperatures above 35 K, the total asymmetry undergoes a negligibly small relaxation, signalling lack of static magnetic order (Fig. 4a). In the time spectra observed in zero field (ZF), a fast decaying front end begins to develop around 35 K, and becomes more pronounced for lower temperatures. This early time decay results from the build up of static internal field, since a small longitudinal field (LF), *μ*_0_H=0.01 T, eliminates this relaxation via the decoupling effect (Fig. 4b). The time spectra in ZF are fitted with the relaxation function *G*(*t*), expected for a Lorentzian distribution of local fields^16^:


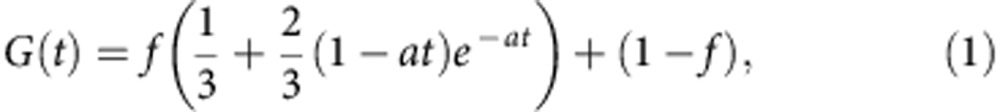


where *f* represents the volume fraction with static magnetic order. Detailed reasoning for the choice of this fitting function is given in the Supplementary Note 2. The temperature dependence of the relaxation rate *a* and the magnetic volume fraction *f* are shown in Fig. 4c. A reasonably sharp transition occurs below *T*_N_=36 K to a state with 100% ordered volume, preceded on cooling by a small temperature region around *T*_N_ characterized by the finite volume fraction *f*, which suggests co-existence of ordered and paramagnetic volumes in real space via phase separation.

An important feature found in both ZF and LF time spectra is the absence of dynamic relaxation, expected for critical slowing down of spin fluctuations around *T*_N_. Such an effect should have resulted in the 1/*T*_1_ relaxation of the asymmetry measured in *μ*_0_*H*=0.01 T, since this LF can eliminate the effect of static magnetism, while dynamic effects survive in a small LF. The observed relaxation rate 1/*T*_1_ in LF=100 G was <0.02 μs^−1^. Using the well-known formula 1/*T*_1_∼Δ^2^*τ*_c_, with the local field strength Δ=1 μs^−1^, we then find that the correlation time *τ*_c_ of the local field fluctuations should be <20 ns. Critical slowing down of spin fluctuations slower than this should have resulted in an observable decay in the LF spectra near the spin freezing temperature ∼36 K. Another piece of information comes from the absence of the decay of the 1/3 component in ZF and the decoupled time spectra in LF at low temperatures. These spectra indicate that the local field at low temperatures in the ordered state is quasi-static, with the time scale of 10 μs or more. See ref. 16 for more details.

Together with the ZF relaxation function (equation (1)) which solely involves static effects, the present data indicate complete absence of dynamic critical behaviour. Although occurring in a limited temperature region, the aforementioned phase separation indicates that the transition is likely first-order, without dynamic critical behaviour. As shown in Supplementary Table 1, similar absence of dynamic critical behaviour associated with phase separation was observed in μSR studies of the itinerant helimagnet MnSi in an applied pressure of 13–15 kbar^17^, near the pressure-tuned quantum crossover to the paramagnetic phase. Such tendencies were also seen in the itinerant ferromagnet (Sr,Ca)RuO_3_ close to the disappearance of static magnetic order around a Ca concentration of 0.7 (ref. 18). The first-order transition may be a generic feature of weak magnetic order in itinerant–electron systems^19^.

As Supplementary Table 2 shows, the magnitude of the internal magnetic field in TiAu in the ordered state is very small, compared with μSR results in other itinerant–electron systems, dilute alloy spin glasses or the incommensurate SDW system (Sr_1.5_Ca_0.5_)RuO_4_ (ref. 20). Although this indicates a very small ordered moment in TiAu, it is not possible to estimate the moment size since the hyperfine coupling constant could depend strongly on the assumption of the location of muon sites. The line shape of the ZF μSR data in equation (1) is obtained for the case of dilute alloy spin glasses where the local field at the muon site varies due to different distances to the moment site^16^. However, the same line shape was also observed in (Sr_1.5_Ca_0.5_)RuO_4_ in which incommensurate SDW order was recently confirmed by neutron scattering. Therefore, it is difficult to determine the spin structure of the TiAu system from the present μSR data alone. In general, the observation of long-lived oscillations by μSR can indicate homogenous long-range order, but the absence of oscillations does not rule out long-range magnetic correlations. This feature can be found in many cases of known AFM and FM systems, as reviewed in the Supplementary Note 2.

### Evidence of Long-range antiferromagnetic order

Neutron diffraction measurements above (*T*=60 K) and below (*T*=2 K) the ordering temperature reveal a resolution-limited magnetic peak (inset, Fig. 4d, Supplementary Fig. 3) in the low temperature data. The temperature dependence of this peak indicates magnetic ordering at *T*_N_=36(2) K. The magnitude of the ordered moment is estimated to be 0.15 μ_B_ per Ti, consistent with a small itinerant moment. The neutron data eliminate the possibility that the observed magnetism is due to dilute magnetic impurities. The μSR results that show a magnetic phase fraction of 100% eliminate the possibility of neutron signal coming from a minority phase of small volume. These arguments suggest that the magnetism of the present system is an intrinsic feature of TiAu. Since it is not possible to determine the origin of the LSG line shape observed in μSR (see Supplementary Figs 4 and 5 and the discussion in Supplementary Note 2), the present work relies on neutron diffraction measurements to show long-range order, and μSR for information on volume fractions and absence of critical spin dynamics. More detailed and general discussions on limitations of μSR and neutron measurements, and the merits of the combination of the two methods, can be found in Supplementary Note 3, with the Supplementary Fig. 6 illustrating various spin patterns possible in real compounds, and Supplementary Fig. 7 showing possible neutron (a–f) and μSR (g–j) responses.

### Band structure calculations

Even though the experimental evidence demonstrates long-range antiferromagnetic, small moment ordering in orthorhombic TiAu, a comparison between the experimental data with theoretical results from band structure calculations are of interest. These were performed using a full-potential DFT^21^ while taking spin–orbit coupling into account (see the Methods section for more details). A number of possible magnetic configurations were considered: ferromagnetic (FM), AFM SDW with modulation vectors **Q**_1_=(0, 2*π*/3*b*, 0) (AFM1) and **Q**_2_=(0, *π*/*b*, 0) (AFM2). Their energies relative to the paramagnetic state were estimated to be *E*_FM_=−35 MeV per Ti, *E*_AFM1_=−47 MeV per Ti and *E*_AFM2_=−34 MeV per Ti, respectively. Given the systematic error bars of the exchange-correlation potential employed in the DFT, the calculated energy values point to a AFM ground state with wavevector **Q**_exp_=(0, *k*, 0), with *k* between 2*π*/3*b* (AFM1) and *π*/*b* (AFM2), a value consistent with the neutron diffraction experiments. However the uncertainty in determining the exact wavevector from DFT does not affect the conclusions from the overwhelming experimental evidence for the itinerant AFM order in TiAu. Furthermore, the calculation yields a small ordered magnetic moment *μ*_calc_ for all surveyed configurations, 0.52 μ_B_ per Ti≤*μ*_calc_≤0.74 μ_B_ per Ti, reflecting the itinerant nature of the ordered moment. This DFT overestimate compared to the experimental moment estimate of 0.15 μ_B_ per Ti from neutron scattering is likely a result of strong spin fluctuations which cannot be accounted for by the calculations. The same problem was also encountered in Fe pnictides, which are also itinerant SDW AFM compounds^22^,^23^. In the case of TiAu this may be remedied by future dynamical mean-field theory calculations, beyond the scope of this mainly experimental report of IAFM TiAu.

It is instructive to analyse the origin of magnetism in TiAu using the input from the band structure calculations. A picture that is often employed is that of the weak-coupling random phase approximation, resulting in the generalized Stoner criterion for the bare magnetic susceptibility *χ*^(0)^ at a reciprocal wavevector **Q**^24^:


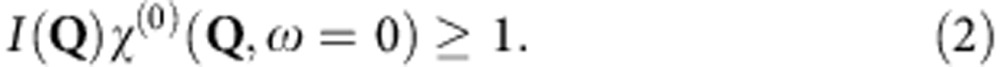


**Figure 5:**
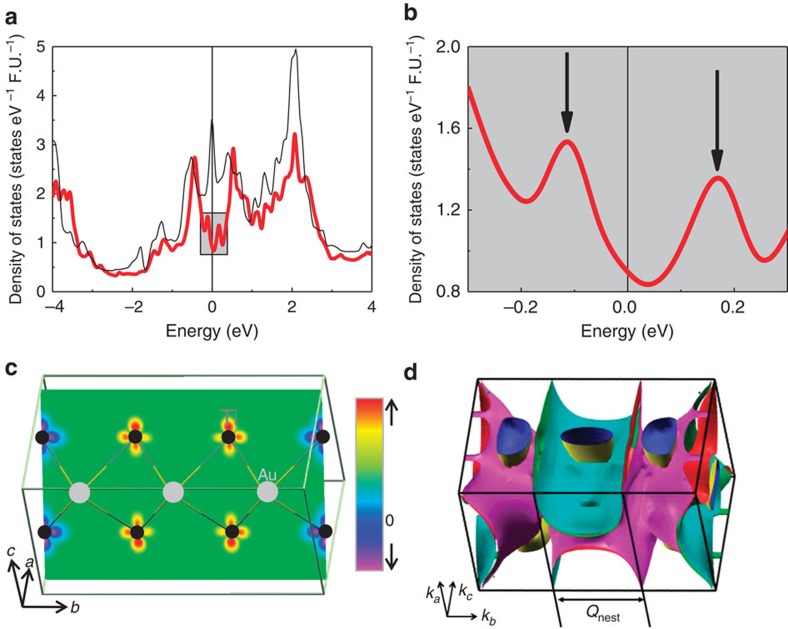
Band structure calculations for TiAu. (**a**) The non-magnetic density of states (thin black line) exhibits a peak close to the Fermi surface, similar to that seen in other itinerant magnets. (**b**) For the AFM1 ground state, the finite total density of states (thick red line) at the Fermi energy is flanked by two peaks around 0.1 eV, which explains the metamagnetic transition at low *T* (see text). (**c**) The electron spin density shows a modulation along the *b* axis, consistent with the *k*=2*π*/3b nesting shown in **d**. The colour scale indicates degree of electron polarization, ranging from spin-up (red) to spin-down (purple). The arrows identify the crystallographic directions, marked by *a*, *b* and *c* axes. Fermi surface with nesting vector *Q*_*calc*_=(0,2*π*/3*b*,0) is shown in **d**. The Fermi surface is coloured for ease of viewing. The arrows identify the wavevectors in the reciprocal space, marked by *k*_a_, *k*_b_ and *k*_c_.

Within this picture, the idealized itinerant limit can be understood as the case where *χ*(**Q**) is strongly peaked at a particular **Q** vector, resulting in a SDW ordering at that wavevector. In this case, the **Q** dependence of the interaction strength *I*(**Q**) is unimportant. Traditionally, for instance in Cr^25^, the peak in *χ*^(0)^(**Q**) is understood as originating from Fermi surface nesting. Indeed, the calculated Fermi surface of the non-magnetic TiAu (Fig. 5c and Supplementary Fig. 8) exhibits large nearly nested regions in the *k*_b_ direction with the nesting wavevector **Q**_nest_=(0, *k*, 0) discussed above. In one spatial dimension, nesting is known to result in a logarithmic divergence of the susceptibility at **Q**_nest_=2*k*_F_, *χ*^(0)^(**Q**_nest_)∼−*ρ*(*E*_F_)log[*ρ*(*E*_F_)*T*], resulting in the celebrated Peierls mechanism for charge density wave and SDW, with *ρ*(*E*_F_) representing the electronic density of states at the Fermi level. However in higher dimensions, it was pointed out that the divergence of *χ*^(0)^ is strongly suppressed^26^, and moreover, the ordering wavevector **Q** of the charge density wave (and by analogy, SDW) does not generally coincide with the nesting wavevector **Q**_nest_^27^, shown to be the case in example, rare earth tritellurides^26^,^27^. The reason behind this is that the real part of the susceptibility *χ*(**Q**) contains the integration over all bands deep below the Fermi level, whereas nesting is the property of the Fermi surface itself and its effect is limited (except in the special case of perfect nesting, as is the case in one dimension).

It is a non-trivial task to calculate *χ*^(0)^(**Q**) accurately from the DFT results, however the fact that all three magnetic configurations considered above have comparable energies indicates that *χ*^(0)^(**Q**) is not a simple single-peaked function. This goes to show that, while magnetism in TiAu is close to the itinerant limit, its mechanism is more complicated than in Cr^25^. The difference between Cr and TiAu is further highlighted by the fact that the latter has a considerably larger drop in the relative magnetic susceptibility Δ*M*/*M* at *T*_N_, where 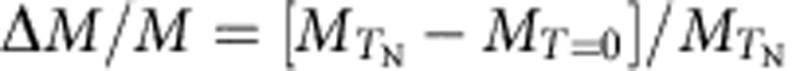. In TiAu, Δ*M*/*M* is ∼20%, nearly five times larger than in Cr^25^. In the latter, the small magnetization decrease at *T*_N_ had been attributed to the small spin susceptibility (and not the larger orbital component) being affected by the gap associated with the SDW transition. Conversely, the larger magnetization change in TiAu might indicate a sizable effect on the orbital magnetization, as the SDW transition is now associated with more two dimensional nesting than that in Cr.

## Discussion

We evidence that orthorhombic TiAu is an IAFM without local moments and analogous to the only two IFMs with no magnetic elements, Sc_3_In and ZrZn_2_. Experimental evidence for the itinerant character of the magnetic state in TiAu includes small magnetic moment in the ordered state compared to the paramagnetic moment, as well as small magnetic entropy at *T*_N_. It is readily apparent that strong spin fluctuations are at play in this magnetic system. The exact role of the spin fluctuations, their strength, as well as the details of the magnetic structure in the ordered state, remain to be fully elucidated with further experiments. Additionally, doping experiments are underway, indicating that the magnetic order in doped TiAu is suppressed to 0 in a quantum critical regime with strong spin fluctuations. More detailed calculations using dynamical mean-field theory, left to a future in-depth theoretical study on the IAFM. Ultimately, the search for IAFMs appears to be a promising avenue for furthering our understanding of the complex magnetism, and providing the unifying picture for local and itinerant moment magnetism.

## Methods

### Sample preparation and composition analysis

Polycrystalline samples of TiAu were prepared by arcmelting, with mass losses no more than 0.3%. The hardness of the arcmelted samples rendered powder X-ray diffraction experiments difficult, and therefore X-ray diffraction data were collected at room temperature off the cross-section (about 3 mm in diameter) of cut and polished specimens using a custom 4-circle Huber diffractometer with graphite monochromator and analyser in non-dispersive geometry, coupled to a Rigaku rotating anode source producing CuKα radiation. X-ray photoemission spectroscopy was performed on the polished surface of the TiAu sample, using an XPS Phi Quantera spectrometer with a monochromatic Al X-ray source and Ar ion sputtering gun, used to cleanse the surface of contaminants. The alignment was checked by comparing the binding energy of the C1*s* peak to the published one^28^. More details can be found in the Supplementary Note 1. Heat treatment (annealing) at several different temperatures resulted in no measurable changes in either the structural or physical properties.

### Magnetization, specific heat and resistivity measurements

The d.c. magnetization was measured in a Quantum Design (QD) Magnetic Property Measurement System from 2 to 400 K. At temperatures above 400 K, the magnetization data were collected using the Vibrating Sample Magnetometer option of a QD Physical Property Measurement System (PPMS) equipped with an oven. Specific heat using an adiabatic relaxation method, and four-probe d.c. resistivity measurements from 2 to 300 K were carried out in the QD PPMS environment.

### μSR measurements

ZF and LF (up to *μ*_0_*H*=0.01 T) μSR measurements were performed at the M20 channel of TRIUMF, Vancouver, Canada. The cut TiAu samples of thickness around 0.1 cm and area of about 3.5 cm^2^ were mounted on a silver foil and oriented perpendicular to the direction of the incoming muons. For all experiments the beam momentum was ∼28 MeV c^−1^, with muons polarized along the flight direction.

### Neutron diffraction measurements

Neutron diffraction data were collected on the BT-1 powder diffractometer and BT-7 thermal triple-axis spectrometer at the NIST Center for Neutron Research. For the neutron diffraction measurements the sample was sealed with helium exchange gas and mounted in a closed cycle refrigerator with a base temperature of 2.5 K. To search for magnetic scattering the high intensity/coarse resolution BT-7 spectrometer was employed in two-axis mode, with a fixed initial neutron energy of 14.7 MeV (*λ*=2.369 Å) and collimator (full-width-half-maximum) configuration open—PG(002) monochromator—80′—sample—80′ radial-collimator—position-sensitive detector^29^. Supplementary Fig. 3 shows the observed counts (full circles) for the magnetic peak. The solid curve is a fit to Gaussian (instrumental) peaks (solid curve). To characterize the sample and search for possible structural changes associated with the magnetic phase transition the BT-1 high resolution powder diffractometer was used. Collimators of 15′, 20′ and 7′ were used before and after the Cu (311) monochromator (*λ*=1.5401 Å) and after the sample, respectively, and data were collected in steps of 0.05° in the 2*θ* range of 3–168°. BT-1 data were used for the crystallographic analysis at *T*=5 K (black) as shown in Supplementary Fig. 2, with all peaks identified as the orthorhombic *Pmma* TiAu phase (vertical marks). The results of Rietveld structural refinements with the General Structure Analysis System software of the data below (*T*=5 K) and above (*T*=60 K) *T*_N_ as well as those obtained from room temperature X-ray diffraction are summarized in Supplementary Table 1.

### Theoretical calculations

Band structure calculations were performed using the full-potential linearized augmented plane-wave method implemented in the *WIEN2K* package^30^. The PBE-GGA was used as the exchange potential, the default generalized gradient approximation for the exchange-correlation potential in *WIEN2K*^31^ and spin–orbit coupling was included in a second-variational fashion^32^. The lattice parameters and atomic positions were determined from both neutron and X-ray diffraction and are reported in the Supplementary Table 1. A 10 × 10 × 10 *k*-point grid was used, and shift away from high symmetry directions was allowed. The convergence criterion for force is 1 mRyd per a.u. (1 Ryd=13.6 eV), with the residual force for the **Q**=(0, 2*b*/3*π*, 0) state less than 3.5 mRyd per a.u. (or 90 MeV Å^−1^). For the density of states plot, the Gaussian broadening was used, with a broadening factor of 3 mRyd. In order to make the Fermi surface plot (shown in Fig. 5c) easier to read, a separated Fermi surface plot for the different bands in shown in Supplementary Fig. 8.

## Additional information

**How to cite this article:** Svanidze, E. *et al.* An itinerant antiferromagnetic metal without magnetic constituents. *Nat. Commun.* 6:7701 doi: 10.1038/ncomms8701 (2015).

## Supplementary Material

Supplementary Information Supplementary Figures 1-8, Supplementary Tables 1-2, Supplementary Notes 1-3 and Supplementary References
