# Supplementary material for: An itinerant antiferromagnetic metal without magnetic constituents
Source: Nat Commun. 2015 Jul 13;6:7701. doi: 10.1038/ncomms8701 (PMC4510670; doi:10.1038/ncomms8701)
Supplement: Supplementary Information — Supplementary Figures 1-8, Supplementary Tables 1-2, Supplementary Notes 1-3 and Supplementary References [file ncomms8701-s1.pdf]

SUPPLEMENTARY INFORMATION

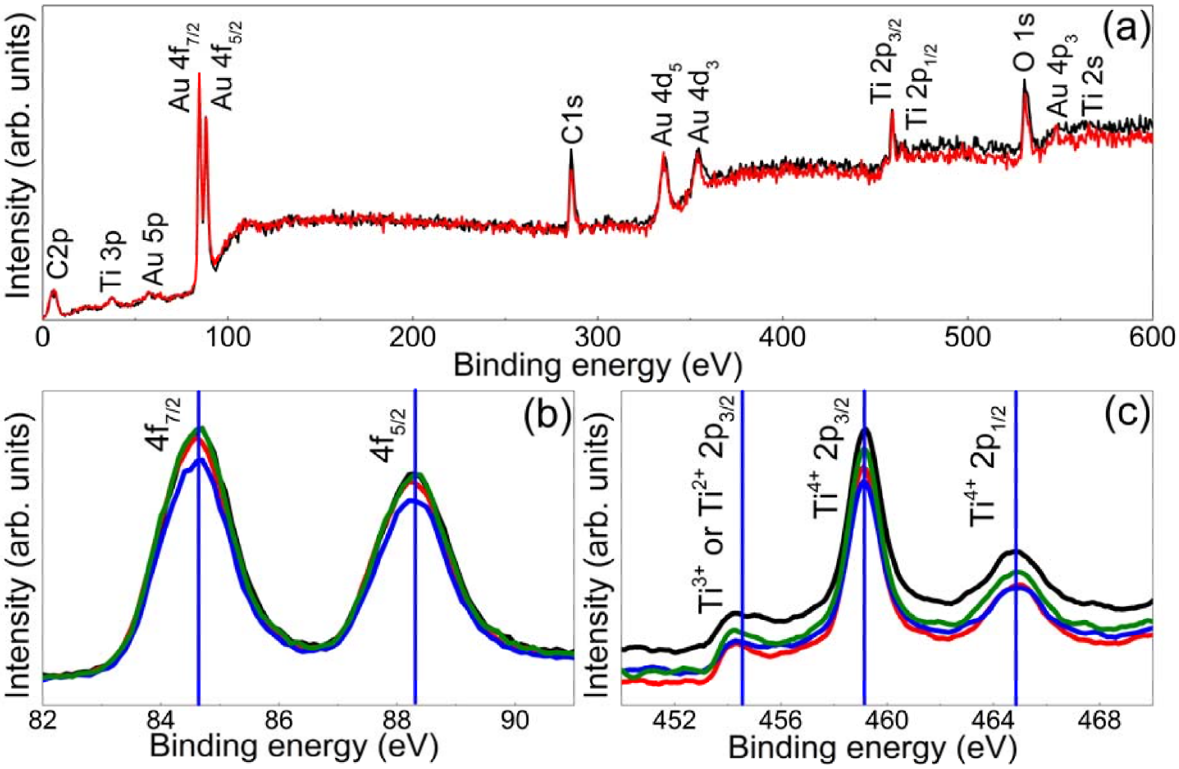

Supplementary Figure 1: **X-ray photoemission spectroscopy.** (a) A survey scan indicates presence of Ti, Au, C and O. The latter two elements are typically present in all samples studied by this technique. The elemental scans for Au (b) and Ti (c) were used to extract valence information (see Supplementary Note 1).

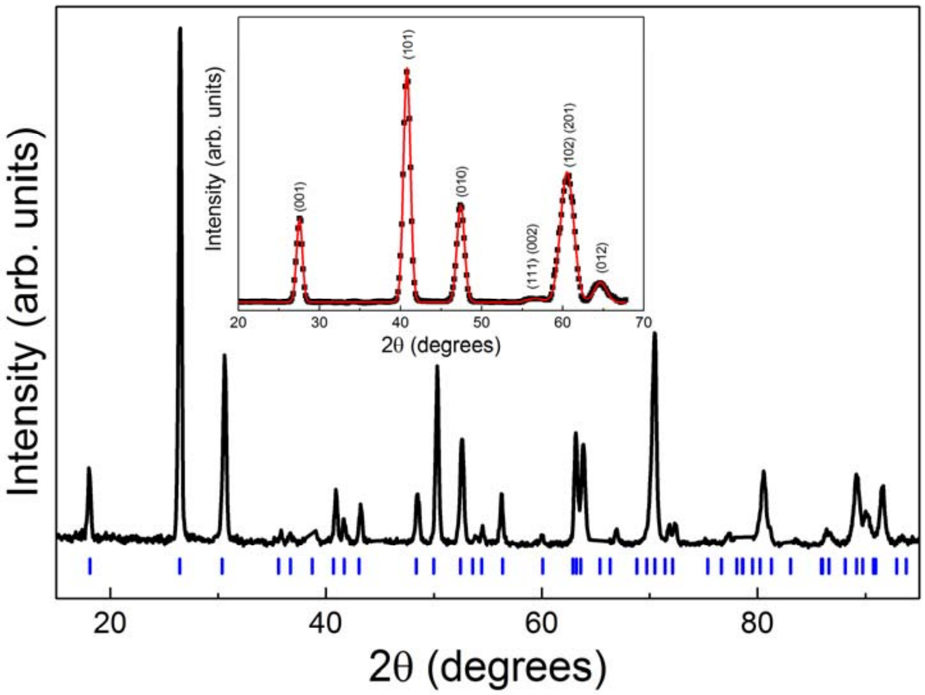

Supplementary Figure 2: **Neutron diffraction data.** A high resolution diffraction pattern for T = 5 K (λ = 1.5401 Å) is indexed with the orthorhombic *Pmma* TiAu phase, denoted by blue vertical marks. The inset shows a portion of the high-intensity diffraction data (full squares) taken on BT-7 at T = 2.5 K (λ = 2.369 Å). Fig. 4 (main text) shows the observed counts (full circles) for the magnetic peak, with the solid curves representing fits to Gaussian (instrumental) peaks.

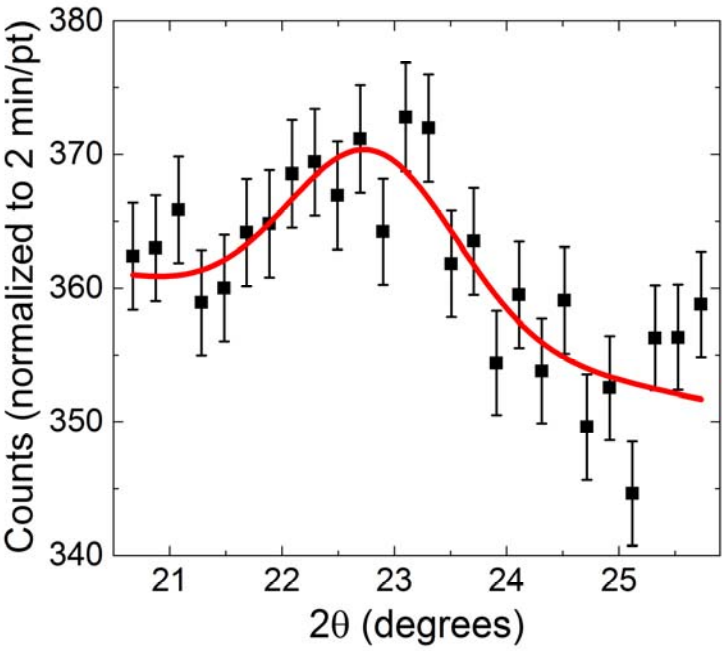

Supplementary Figure 3: **Magnetic peak data at *T* = 2.5 K.** The symbols are measured data, with the Gaussian fit shown as a solid line.

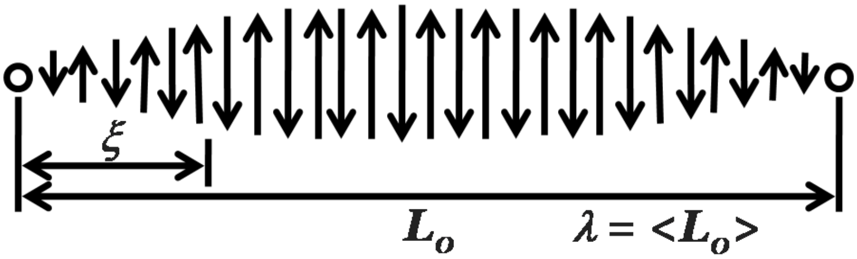

Supplementary Figure 4: **Spin chain.** Quasi one-dimensional *S* = 1/2 spin chain [21].

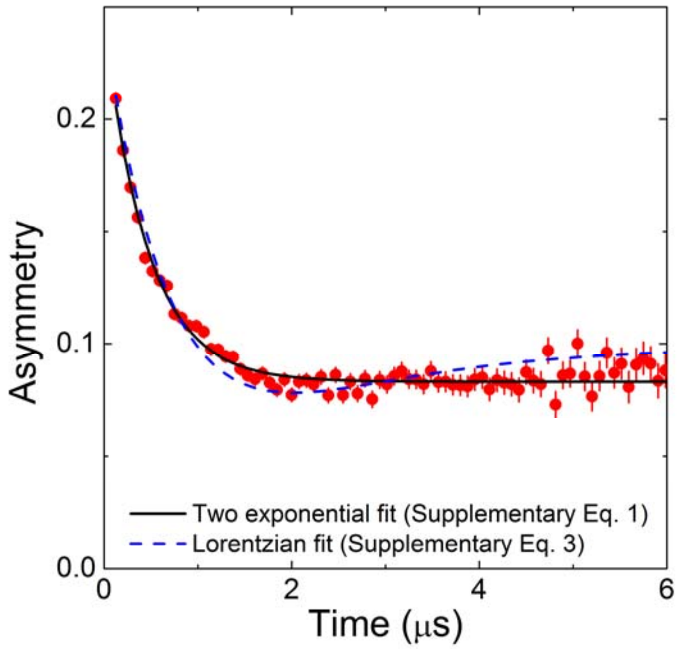

Supplementary Figure 5: **μSR asymmetry analysis.** Comparison of the fit with the LSF line shape (Eq. 3, dashed blue line) and the two-exponential phenomenological line shape (Eq. 1, solid black line). The difference is subtle, and the choice between these two functions does not alter the essential part of the presented conclusions. The reasoning for using LSG function is described in Supplementary Note 2.

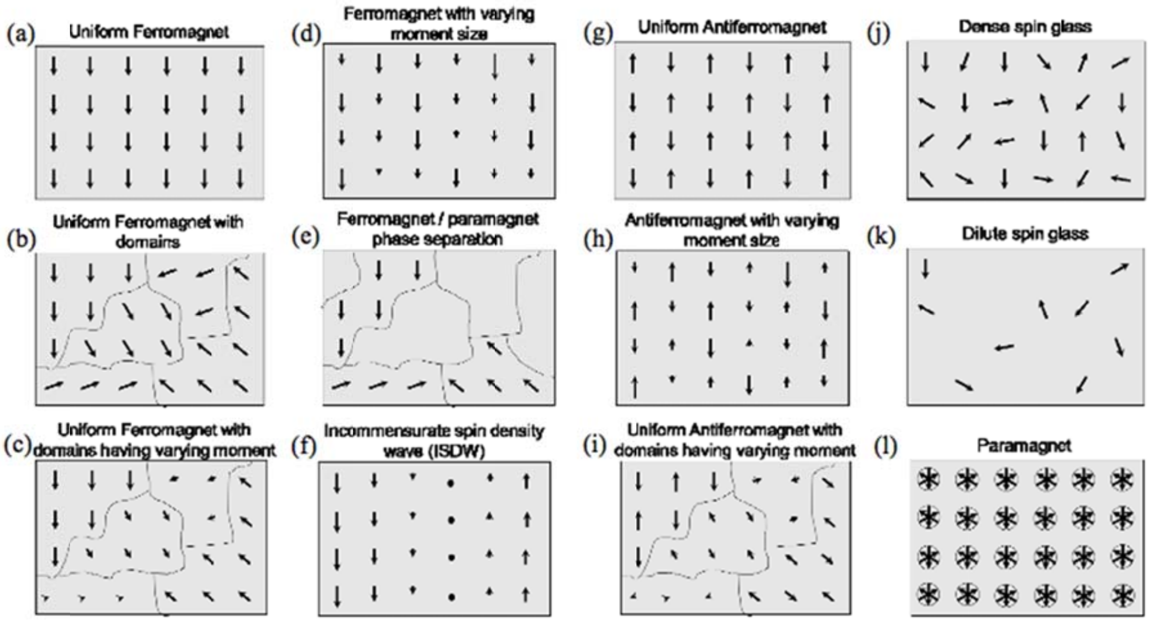

Supplementary Figure 6: Various spin patterns (a) – (l).

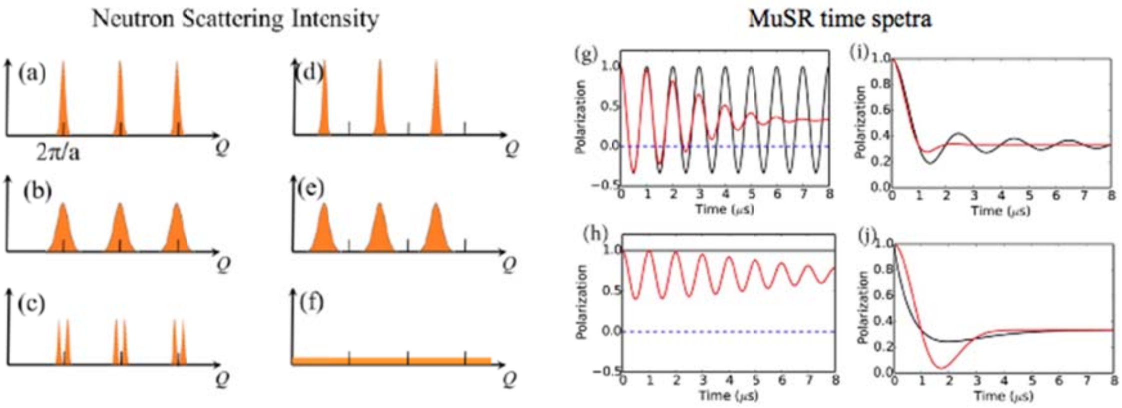

Supplementary Figure 7: Neutron scattering (a) - (f) and μSR responses (g) - (j).

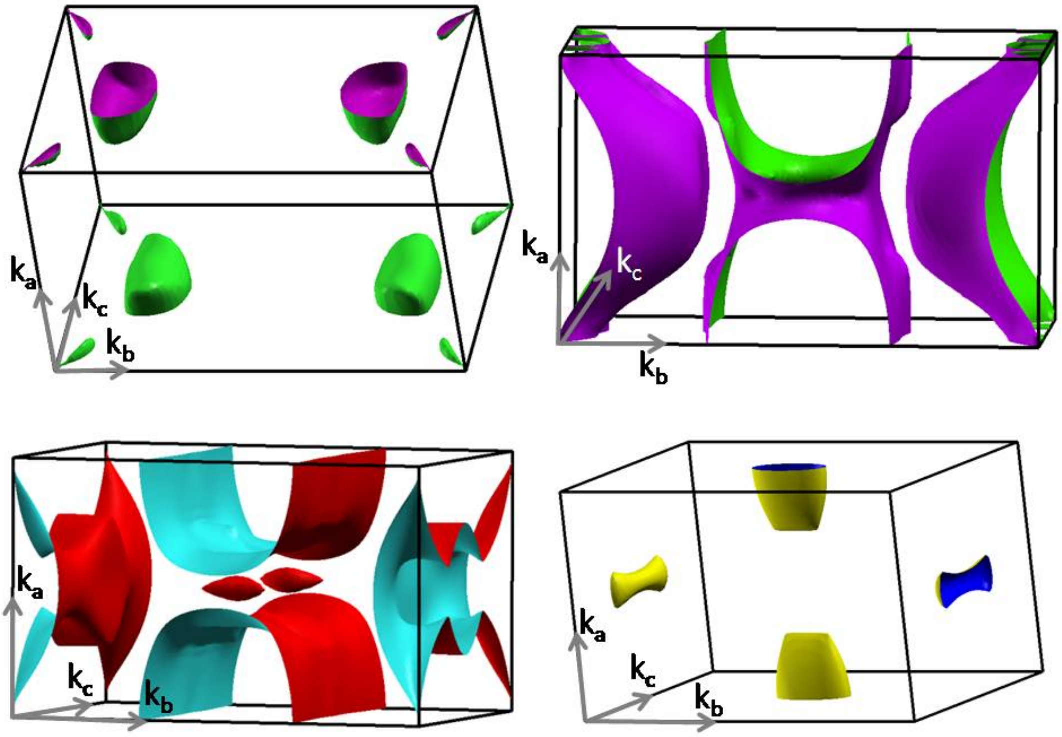

Supplementary Figure 8: **Separated Fermi surface for different bands.** The Fermi surfaces are colored for the ease of viewing. The arrows identify the wavevectors in the reciprocal space, marked by k_a, k_b, and k_c.

Supplementary Table 1: **Crystallographic information for *Pmma* TiAu**.

	Neutron diffraction		X-ray diffraction
Temperature (K)	$T = 5$	$T = 60$	$T = 300$
Lattice parameters			
a (Å)	4.622(1)	4.622(0)	4.632(3)
b (Å)	2.914(5)	2.916(6)	2.948(9)
c (Å)	4.897(0)	4.895(9)	4.885(5)
Atomic positions			
Ti	(0.250(0), 0.000(0), 0.311(0))	(0.250(0), 0.000(0), 0.308(2))	(0.250(0), 0.000(0), 0.313(3))
Au	(0.250(0), 0.500(0), 0.817(6))	(0.250(0), 0.500(0), 0.817(6))	(0.250(0), 0.500(0), 0.820(2))

Supplementary Table 2: Comparison of μSR results for itinerant helimagnetic, FM, SDW, SG, and CDW systems.

	Pressure (kbar)	Chemical composition	Relaxation rate ($T = 0$) Δ^a (μs^{-1})	Precession frequency ν (MHz)	Ordered volume ($T = 0$) (%)	Ground state	Ordered moment (μ_B F.U.$^{-1}$)	Transition temperature range (K)	Dynamical critical behavior	Reference
TiAu	0		1		100	AFM SDW		33 - 38	no	present
MnSi	0			12	100	Helical	0.4	29	yes	[14]
	13 - 15			11	20 - 80	Helical	0.4	5, 10	no	[14]
$SrRuO_3$	0		80	15, 30	100	FM	0.7	160	yes	[15]
(Sr, Ca)RuO_3	0	$Ca_{0.6\text{-}07}$	20		40 - 60	FM	0.1 - 0.2	25	weak	[15]
(Sr, Ca)RuO_4	0	$Ca_{1.5}Sr_{0.5}$	8		100	IC-SDW	0.2 - 0.3	8	yes	[8]
Cu(Mn)	0	1 % Mn	10		100	SG	0.04 (4/Mn)	10	yes	[22]
$BaTi_2(As,Sb)_2O$	0		0.1 - 0.2 (NDB)[b]			CDW[c]				[24]

[a] Generally Δ scales as 2πν
[b] NDB: nuclear dipolar broadening
[c] Recently confirmed (unpublished)

Supplementary Note 1

X-ray photoemission spectroscopy (XPS) is a technique extremely sensitive in resolving the elemental composition [1]. O and C peaks can often be present in XPS spectra [1], a consequence of sample preparation. The rest of the peaks in Supplementary Figure 1 correspond to Ti and Au, confirming the purity of the 1:1 *Pmma* phase, consistent with neutron (below) and x-ray (not shown) diffraction data. XPS is also employed in determining the valence of elements in many compounds [2-5]. Supplementary Figure 1b reveals that the binding energy of $4f_{7/2}$ Au is close to 85 eV, suggesting that Au is close to a Au^{1+} state [6]. The large energy absorption of Ti does not allow for high resolution measurements, limiting the number and quality of peaks that can be successfully analyzed. In TiAu, the binding energy for the most pronounced Ti $2p_{3/2}$ line is split into two peaks, one at ≈ 455 eV (for Ti^{2+} or Ti^{3+}) [7] and another one at ≈ 459 eV (Ti^{4+}) [7], as shown in Supplementary Figure 1c. A comparison of the areas under the respective curves suggests the valence of Ti to be 3.8 ± 0.12. In this itinerant magnet, the XPS results showing a small d electron contribution are consistent with the small itinerant moment ordering indicated by μSR and neutron measurements (main text), and rule out the presence of a local moment in TiAu.

Supplementary Note 2

In the ordered state of prototypical ferromagnets (FMs) or antiferromagnets (AFMs) at low temperatures, long-lived sinusoidal muon spin precession has been observed with more than several oscillation periods in ZF μSR. Good examples for those can be found in AFM Ca_2RuO_4 (T_N = 110 K) ([8], supplementary info A), La_2CuO_4 (T_N = 240 K) [9], $BaFe_2As_2$ (T_N = 140 K) [10], (CuBr)$LaNb_2O_7$ (T_N = 35 K) [11], Ca_2CuO_3 (T_N = 10 K) [12], Sr_2CuO_3 (T_N = 5 K) [12], and helical magnet MnSi (T_C = 29 K) [13]. Bessel function line shapes with long-lived oscillations were observed in the incommensurate spin density wave (IC-SDW) system $(TMTSF)_2PF_6$ [14] and for the stripe magnetic order of $(La, Ba)_2CuO_4$ [9]. Observation of homogeneous internal fields can certainly indicate long-range spatial spin order.

However, there are also many cases of known long-range ordered magnetic systems in which muon spin precession is either heavily damped or even absent. Examples for these cases include FM systems (Sr,Ca)RuO_3 [14,15], $(Sc, Lu)_3In$ [16], IC-SDW systems $CeCu_2Si_2$ (α - phase) [17, 18], $(Sr_{1.5}Ca_{0.5})RuO_4$ [8,19], and $Sr_2(Ru, Ti)O_4$ [8, 20], as well as low dimensional and/or frustrated magnetic systems such as $Sr_2(Ca, Pd)O_3$ [21], Cu(Cl, Br)$LaNb_2O_7$ and CuClLa$(Nb, Ta)_2O_7$ [22]. In general, the absence of oscillation or over-damped line shapes could results from several reasons including: (a) multiple muon sites, (b) inhomogeneous fields from magnetic domain boundaries and/or crystallographic grain boundaries, as well as (c) spatial variation of the ordered moment size in long-range ordered systems. An example for the case (c) is discussed in Ref. [21] for $Sr_2(Cu, Pd)O_3$, where Pd atoms generate spatial inhomogeneity in the ordered moment size of the quasi one-dimensional S = 1/2 Cu spin chain, as illustrated in Supplementary Figure 4.

In TiAu, the initial fit of the muon spin relaxation data includes two simple exponential relaxing components, yielding the black curve shown in Supplementary Figure 5. The corresponding asymmetry function is of the following form:

$$A(t) = A(fe^{-\lambda_s t} + (1-f)e^{-\lambda_f t}) \qquad (1)$$

However, considering the polycrystalline nature of our sample, the isotropy of the overall local magnetic field dictates an average of 1/3 of all muons to have spin parallel to the local field, hence showing no relaxation arising from the random static magnetic field in the sample. In a system with randomly oriented, dense, and static magnetic moments, a Gaussian Kubo-Toyabe (GKT) relaxation function [23] is usually expected:

$$A_{Gaussian\,KT}(t) = A\left(\frac{1}{3} + \frac{2}{3}(1 - \sigma^2 t^2)e^{-\frac{1}{2}\sigma^2 t^2}\right) \qquad (2)$$

while in dilute spin systems a corresponding Lorentzian spin-glass (LSG) function, initially developed [22] for dilute alloy SGs CuMn and AuFe, is due to the Lorentzian internal field distribution [12]:

$$A_{Lorentzian\,SG}(t) = A\left(\frac{1}{3} + \frac{2}{3}(1 - at)e^{-at}\right) \qquad (3)$$

For the polycrystalline TiAu sample a LSG function (blue line, Supplementary Figure 5) is more appropriate and yields a distinctively better fit than the Gaussian Kobo-Toyabe function. This might give an impression that magnetic order of TiAu is associated with very dilute ordered moments. However, we would like to remind here that both in FM systems (Ca,Sr)RuO_3 and IC SDW systems ($Sr_{1.5}Ca_{0.5}$)RuO_4, which have a decent density of ordered Ru moments, the LSG line shape was observed over a large doping range near the disappearance of the static magnetic order [8,14,15]. Furthermore, this line shape also fit well the 5 % Mn-doped CuMn SGs [22], in which more than one Mn moment exists in every 5 unit cells of the FCC Cu crystal structure. In the FCC structure, one atom belongs to 8 unit cells. Therefore, in 5 % CuMn, most of the Mn spins have their nearest neighbor Mn spin within these 8 unit cells. In this sense, the observation of the LSG line shape is not restricted to just dilute spin systems.

As shown in Fig. 5, the two-exponential function (Supplementary Eq. 1) gives a slightly better fit than the LSG function (Supplementary Eq. 3). This is partly due to the fact that Supplementary Eq. 1 has more freedom than the Supplementary Eq. 2. For the estimate of the magnetic volume fraction f, it is important to use a theoretically derived LSG function which properly accounts for the 1/3 component in ZF. There is no theoretical field distribution which can generate the sum of two exponential function Supplementary Eq. 1. In view of these two limitations of Supplementary Eq. 1, we used the LSG function (Eq. 1 and Supplementary Eq. 3) for the present analysis.

Even for systems which exhibit Bragg peaks in neutron diffraction due to long-range magnetic order, if there is spatial distribution in the ordered moment size, then the μSR line shape can exhibit fast damping or absence of oscillations. This could explain the absence of oscillations in the ZF μSR data [8] in the IC-SDW systems ($Sr_{1.5}Ca_{0.5}$)RuO_4 [19] and Sr($Ti_{0.09}Ru_{0.91}$)O_4 [20]. Therefore, the observation of an AFM Bragg peak in neutron scattering and the over-damped LSG line shape in μSR can be reconciled within the picture of long-range (neutrons) static (μSR)

magnetic order. Positive muon is a local probe whose information is integrated over momentum space. The determination of long-range magnetic order can be done better by neutron scattering, as clearly stated in the main text of the present paper.

However, μSR results reflect signals from all the muons in the total sample volume, with the information about the volume fraction of the magnetically ordered region. In μSR studies, the local field at the muon site is generated mainly via dipolar interactions of surrounding magnetic moments. If an ordered moment of 1 μ_B is 1 Å away from a muon site, it will generate a local field of 1 T. When the distance is 3 Å, the local field becomes about 400 G. For the distance of 10 Å, the field becomes 10 G. The randomness of the internal magnetic field of 10 G will result in muon spin relaxation rate of about 1 μs^{-1} in ZF. Therefore, the observation of relaxation in the ordered state in TiAu, with the relaxation rate of 1 μs^{-1} in the full volume fraction can rule out the existence of paramagnetic or non-magnetic domains larger than 10 - 15 Å in size with more than ~ 5 % of the volume fraction.

ZF μSR spectra with overdamped or absent oscillations have been often observed in various magnetic systems near the disappearance of magnetic order and/or at a quantum critical point. On the other hand, many of the above-mentioned examples involve built-in randomness due to chemical substitutions. TiAu is synthesized with nominal 1:1 stoichiometric composition. Further detailed studies of the origin of the observed LSG line shape and local field distributions in TiAu could lead to more insight on the generic behavior of homogeneity near a magnetic quantum critical point. With the currently available data, however, it is impossible to determine the origin of the LSG line shape. Thus, we rely on the neutron scattering information for discussions of the long-range magnetic order, and μSR for discussions of the volume fraction of magnetic order.

Supplementary Note 3

To understand the limitations of neutron scattering and μSR as probes for magnetic systems and merits to combine the two methods, here we consider responses of neutrons and muons for 12 spin patterns listed in Supplementary Figure 6. For simplicity, here we discuss the case for isotropic polycrystalline (or powder or ceramic) specimens, which corresponds to the present study of TiAu. The uniform ferromagnet in Supplementary Figure 6a generates sharp Bragg peaks as seen in Supplementary Figure 7a at the momentum transfer $Q = 2\pi/a$, where a denotes the lattice constant. The formation of domains and/or short range order broadens the Bragg peaks as shown in Supplementary Figure 7b. If different domains have different ordered moment sizes like in Supplementary Figure 6c or 7d, the pattern in Supplementary Figure 7b is superimposed with an incoherent signal (Supplementary Figure 7f). When the system is phase separated with a large paramagnetic volume like in Supplementary Figure 6e, the neutron pattern in Supplementary Figure 7a or Supplementary Figure 7b will appear with reduced intensity. For an IC-SDW in Supplementary Figure 6f, satellite Bragg peaks as in Supplementary Figure 7c will be observed. For a uniform AF in Supplementary Figure 6g, the Bragg peaks will shift to $Q = [\pi/a + n*2\pi/a]$ with n = integer. For long-range AF with spatial variation of ordered moment size seen in Supplementary Figure 6h, the sharp Bragg peaks of Supplementary Figure 7d are superimposed by the incoherent signal in Supplementary Figure 7f. For the case of an AF with domains having widely varying ordered moment size (Supplementary Figure 6i), the neutron

pattern in Supplementary Figure 7e will be superimposed with that in Supplementary Figure 7f. For the dense (Supplementary Figure 6j) and dilute (Supplementary Figure 6k) spin glasses, neutron intensity will be spread over the momentum space as shown in Supplementary Figure 7f. For paramagnets undergoing fast fluctuations (Supplementary Figure 6l), the neutron intensity is spread as in Supplementary Figure 7f and will be minimal for measurements with sufficient energy resolution. In the present case of TiAu, where the moment size is very small, it is impossible to detect any incoherent response (Supplementary Figure 7f). Therefore, the sharp antiferromagnetic Bragg peak can imply the spin pattern of Supplementary Figure 6g and Supplementary Figure 6h, and somewhat broadened Bragg peak can imply pattern in Supplementary Figure 6i as well as the phase separation (Supplementary Figure 6e) between antiferromagnetic and paramagnetic regions. It is impossible to distinguish these by neutron study alone.

For μSR, one would expect a non-oscillating signal for a paramagnet with the $1/T_1$ exponential relaxation. For fast spin fluctuations, small instantaneous moment size, and absence of large nuclear dipolar field, one expects no relaxation, akin to the black line in Supplementary Figure 7h, as was observed in TiAu above T_N. Uniform ferromagnetic or antiferromagnetic order (Supplementary Figure 6a and g) will often generate a long-lived muon spin precession in ZF, as shown in the time spectrum by the black lines in Supplementary Figure 7g. Domain formation and short-range order will cause fast damping of this signal like the red line in Supplementary Figure 7g. Large variation of local moment size in ferro- or antiferromagnetic order, shown in Supplementary Figure 6c, d, h, and i will lead to a wide distribution of static internal fields, and generate time spectra close to the LSG function shown by the black line in Supplementary Figure 7j originally obtained for dilute spin glasses (Supplementary Figure 6k). IC-SDW generates Bessel function lineshape (black line in Supplementary Figure 7i), but additional broadening of the local field will lead to a damped Bessel function (red line in Supplementary Figure 7i) which is often indistinguishable from LSG lineshape. The spatial phase separation like Supplementary Figure 6e will lead to a distinctly different pattern which is the sum of paramagnetic response and oscillation/relaxation line shapes weighted average of their volume fractions, as shown by the red line in Supplementary Figure 7h. A dense spin glass will result in GKT function (red line in Supplementary Figure 7j), which is distinct from LSG function with the quadratic decay in small t and a large “dip” before recovery to the 1/3 asymmetry.

Depending on the variation of the local field strength, the patterns in Supplementary Figure 6c, d, h and i can exhibit a μSR line shape between GKT and LSG functions. Even in uniform long-range ordered systems shown in Supplementary Figure 6a, g or f, multiple frequencies of the sinusoidal (Supplementary Figure 7g) or Bessel (Supplementary Figure 7i) oscillations could be observed by μSR when there are multiple muon stopping sites having inequivalent symmetries or distances from the magnetic lattice.

The present results with an LSG lineshape in TiAu could be due to a dilute spin glass (Supplementary Figure 6k) as well as any of the cases with a large moment size distribution (Supplementary Figure 6c, d, h, i), as well as an IC-SDW (Supplementary Figure 6f) if there is additional field broadening. However, the phase separation (Supplementary Figure 6e) and dense spin glasses can be ruled out, and it is unlikely to expect the observed results for a very-long ranged uniform ferro- or antiferromagnetic order.

In this way, patterns in Supplementary Figure 6g, h, i, e are compatible with the neutron results and Supplementary Figure 6c, d, f, h, i are consistent with the μSR results. The combination would indicate either Supplementary Figure 6h or Supplementary Figure 6i are the most likely patterns for the present magnetic order in TiAu. The observed sharpness of the neutron antiferromagnetic Bragg peak indicates that the domain size is large if the pattern in Supplementary Figure 6i is realized.

Band structure calculations are also used for comparison of the calculated and experimental magnetic properties of TiAu. Supplementary Figure 6 shows a separated Fermi surface plot for the different bands. Partial Fermi surface nesting is observed along the k_b wave vector, a plausible explanation for the observed itinerant antiferromagnetic order in TiAu.

Supplementary References

[1] Chusuei, C. C. and Goodman, D. W. X-ray Photoelectron Spectroscopy (Academic Press, 2002).

[2] Dudy, L. *et. al.* Yb valence change in $Ce_{1-x}Yb_xCoIn_5$ from spectroscopy and bulk properties. *Physical Review B* **88**, 165118 (2013).

[3] Kitagawa, H., Kojima, N. and Nakajima, T. Studies of mixed-valence states in three-dimensional halogen-bridged gold compounds, $Cs_2Au'Au'''X_6$, (X = Cl, Br or I). Part 2: x-ray photoelectron spectroscopic study. *Journal of the Chemical Society, Dalton Transactions* **11**, 3121-3125 (1991).

[4] Marshall, M. S. J., Newell, D. T., Payne, D. J., Egdell, R. G. and Castell, M. R. Atomic and electronic surface structures of dopants in oxides: STM and XPS of Nb- and La-doped $SrTiO_3$(001). *Physical Review B* **83**, 035410 (2011).

[5] Novotny, Z. *et. al.* Ordered array of single adatoms with remarkable thermal stability: Au/Fe_3O_4(001). *Physical Review Letters* **108**, 216103 (2012).

[6] McNeillie, A., Brown, D. H. and Smith, W. E. X-ray photoelectron spectra of some gold compounds. *Journal of the Chemical Society, Dalton Transactions* **5**, 767-770 (1980).

[7] NIST X-ray Photoelectron Spectroscopy Database. National Institute of Standards and Technology, Gaithersburg. *http://srdata.nist.gov/xps/.*

[8] Carlo, J. P. *et. al.* New magnetic phase diagram of $(Sr, Ca)_2RuO_4$. *Nature Materials* **11**, 323-328 (2012).

[9] Savici, A. *et. al.* Muon spin relaxation studies of incommensurate magnetism and superconductivity in stage-4 $La_2CuO_{4.11}$ and $La_{1.88}Sr_{0.12}CuO_4$. *Physical Review B* **66**, 014524 (2002).

[10] Aczel, A. *et. al.* Muon-spin-relaxation studies of magnetic order and superuid density in an tiferromagnetic NdFeAsO, $BaFe_2As_2$, and superconducting $Ba_{1-x}K_xFe_2As_2$. *Physical Review B* **78**, 214503 (2008).

[11] Uemura, Y. J. *et. al.* μSR studies of the frustrated quasi-2d square-lattice spin system $Cu(Cl,Br)La(Nb,Ta)_2O_7$: evolution from spin-gap to antiferromagnetic state. *Physical Review B* **80**, 174408 (2009).

[12] Kojima, K. *et. al.* Reduction of ordered moment and Neel temperature of quasi one-dimensional antiferromagnets Sr_2CuO_3 and Ca_2CuO_3. *Physical Review B* **78**, 1787-1790 (1997).

[13] Takigawa, M. *et. al.* Positive muon spin rotation and relaxation in the helically ordered state of MnSi. *Journal of Physical Society of Japan* **49**, 1760-1767 (1980).

[14] Uemura, Y. J. *et. al.* Phase separation and suppression of critical dynamics at quantum transitions of itinerant magnets: MnSi and $(Sr_{1-x}Ca_x)RuO_3$. *Nature Physics* **3**, 29-35 (2007).

[15] Gat-Malureanu, I. M. *et. al.* Muon spin relaxation and susceptibility measurements of an itinerant-electron system $Sr_{1-x}Ca_xRuO_3$: quantum evolution from ferromagnet to paramagnet. *Physical Review B* **84**, 224415 (2011).
[16] Svanidze, E. *et. al.* Non-Fermi liquid behavior close to a quantum critical point in a ferromagnetic state without f-electrons. *Physical Review X* **5**, 011026 (2015).
[17] Uemura, Y. J. *et. al.* Coexisting static magnetic order and superconductivity in $CeCu_{2.1}Si_2$ found by muon spin relaxation. *Physical Review B* **39**, 4726-4729 (1989).
[18] Stockert, O. *et. al.* Magnetism and superconductivity in the heavy-fermion compound $CeCu_2Si_2$ studied by neutron scattering. *Physica B* **403**, 973-976 (2008).
[19] Kunkemoller, S., Nugroho, A. A., Sidis, Y. and Braden, M. Spin-density-wave ordering in $Ca_{0.5}Sr_{1.5}RuO_4$ studied by neutron scattering. *Physical Review B* **89**, 045119 (2014).
[20] Braden, M. *et. al.* Incommensurate magnetic ordering in $Sr_2Ru_{1-x}Ti_xO_4$. *Physical Review Letters* **88**, 197002 (2002).
[21] Kojima, K. M. *et. al.* Site-dilution in quasi one-dimensional antiferromagnet $Sr_2(Cu_{1-x}Pd_x)O_3$: reduction of Neel temperature and spatial distribution of ordered moment sizes. *Physical Review B* **70**, 094402 (2004).
[22] Uemura, Y. J., Yamazaki, T., Harshman, D. R., Senba, M. and Ansaldo, E. J. Muon-spin relaxation in AuFe and CuMn spin glasses. *Physical Review B* **31**, 546-563 (1985).
[23] Hayano, R. S. *et. al.* Zero- and low-field spin relaxation studied by positive muons. *Physical Review B* **20**, 850-859 (1979).
[24] Nozaki, Y. *et. al.* Muon spin relaxation and electron/neutron diffraction studies of $BaTi_2(As_{1-x}Sb_x)_2O$: absence of static magnetism and superlattice reflections. *Physical Review B* **88**, 214506 (2013).
